# Theta Oscillations Related to Orientation Recognition in Unattended Condition: A vMMN Study

**DOI:** 10.3389/fnbeh.2017.00166

**Published:** 2017-09-04

**Authors:** Tianyi Yan, Yuan Feng, Tiantian Liu, Luyao Wang, Nan Mu, Xiaonan Dong, Zichuan Liu, Tianran Qin, Xiaoying Tang, Lun Zhao

**Affiliations:** ^1^School of Life Science, Beijing Institute of Technology Beijing, China; ^2^Intelligent Robotics Institute, School of Mechatronical Engineering, Beijing Institute of Technology Beijing, China; ^3^Saddle River Day School Saddle River, NJ, United States; ^4^Beijing Royal School Beijing, China; ^5^School of Education, Beijing Normal University Zhuhai Zhuhai, China; ^6^School of Psychological Research, Beijing Yiran Sunny Technology Co., Ltd. Beijing, China

**Keywords:** visual mismatch negativity (vMMN), wavelet analysis, event-related brain potentials (ERPs), time–frequency analysis, theta oscillation

## Abstract

Orientation is one of the important elements of objects that can influence visual processing. In this study, we examined whether changes in orientation could be detected automatically under unattended condition. Visual mismatch negativity (vMMN) was used to analyze this processing. In addition, we investigated the underlying neural oscillatory activity. Non-phase-locked spectral power was used to explore the specific frequency related to unexpected changes in orientation. The experiment consisted of standard (0° arrows) and deviant (90°/270° arrows) stimuli. Compared with standard stimuli, deviant stimuli elicited a larger N170 component (negative wave approximately 170 ms after the stimuli started) and a smaller P2 component (positive wave approximately 200 ms after the stimuli started). Furthermore, vMMN was obtained by subtracting the event-related potential (ERP) waveforms in response to standard stimuli from those elicited in response to deviant stimuli. According to the time–frequency analysis, deviant stimuli elicited enhanced band power compared with standard stimuli in the delta and theta bands. Compared with previous studies, we concluded that theta activity plays an important role in the generation of the vMMN induced by changes in orientation.

## Introduction

Object characteristics affect visual processing. In addition to color, shape and size, orientation is one important element. Furthermore, detecting orientation changes can be vital to survival, especially under unattended condition. This process can be studied using a component of event-related potential (ERP) called mismatch negativity (MMN).

MMN is a reliable indicator of change-detection processing (Näätänen et al., 2007; Fuentemilla et al., 2008). Visual MMN (vMMN) can be elicited by visual oddball tasks (20% deviant stimuli are inserted randomly in a sequence of 80% standard stimuli). Researchers found that the lateral N1b subcomponent (120–200 ms) and P2 component (200–300 ms) were related to vMMN (Czigler et al., 2006; Hietanen et al., 2008). However, the components varied for different electrode sites and tasks (Shtyrov et al., 2013), especially in orientation oddball tasks (Takács et al., 2013). In addition, the N170 component is very common in orientation oddball tasks related to vMMN (Takács et al., 2013).

Recent studies have provided fairly convincing evidence that vMMN can be elicited by changes in many kinds of object characteristics (Stefanics et al., 2014). Czigler et al. (2002) used red-black and green-black checkerboards to elicit vMMN in 220–260 ms. Kimura et al. (2006) also found vMMN within 200 ms using different colors. Facial expression MMN could be obtained approximately 100–400 ms using neutral, happy and sad faces (Zhao and Li, 2006). In addition, Amenedo et al. (2007) investigated the vMMN that exists for motion-direction tasks. For studies of orientation, many researchers used bars as stimuli with different deviations from cardinal directions (vertical and horizontal; Astikainen et al., 2008; Kimura et al., 2009). The results showed that unattended changes in orientation could induce vMMN. Furthermore, changes from cardinal orientations could induce larger vMMN than oblique angles.

Most studies of vMMN were based on ERPs. Many studies have shown that neural oscillations are related to ERP results (Makeig et al., 2002; Fuentemilla et al., 2008). The amplitude of electrophysiological responses can be examined as a function of frequency to understand their oscillatory characteristics as a function of time. Stothart and Kazanina (2013) found that vMMN was associated with an early increase in theta power (75–175 ms post-stimulus) and that during the 450–600 ms post-stimulus interval, deviant stimuli elicited a stronger reduction in non-phase-locked alpha power than did standard stimuli, reflecting an attentional shift following the detection of change. These findings indicated that different oscillatory frequencies were involved in the vMMN. However, the specific frequency related to the vMMN induced by changes in orientation is still unknown.

To sum up, humans are capable of automatically detecting deviant stimuli. In this study, we hypothesized that changes in orientation could induce vMMN. We focused on the cardinal orientations and sought to investigate the role of neural oscillations in the vMMN response. To this end, a simple arrow symbol was used in this experiment; specifically, upright arrows (0°) served as standard stimuli, and arrows with rotations of 90°/270° served as deviant stimuli. We analyzed them based on ERP and electroencephalogram (EEG) oscillatory characteristics. Time-frequency analysis was used to explore the specific frequency related to the automatic detection of directional changes.

## Materials and Methods

### Subjects

Fifteen students attending the Beijing Institute of Technology in China (six females; age range = 20–23 years old) participated in this experiment. All participants were right-handed, had normal or corrected-to-normal vision, and were free of neurological or psychiatric disorders. This study was reviewed and approved by the School of Life Science Ethics Committee, Beijing Institute of Technology. Written informed consent was obtained from each participant after the nature of the study had been explained.

### Stimuli and Procedure

**Figure 1:**
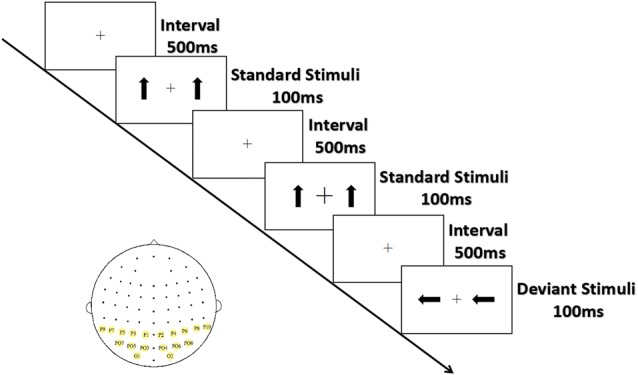
Experimental stimuli and ROIs. Standard stimuli (80%) are upright arrows, deviant stimuli (20%) are arrows with orientations of 90°/270°; sites with yellow background are ROIs.

As shown in Figure 1, the arrows were presented from a viewing distance of 70 cm at a visual angle of 3.68° × 3.42° for 100 ms; they appeared on both sides of a cross that appeared at the center of the screen separated by an inter-stimulus interval (offset-to-onset) of 500 ms. The standards and the deviants were made symmetrical in terms of position about the target area to minimize the effect caused by the tendency to fix gaze away from the central square. Ten standard stimuli were presented at the start of the sequence, and at least two standard stimuli (0° orientation 80% probability) were presented between consecutive deviant stimuli (90° and 270° orientation, 10% probability for each). Three blocks of 300 trials (60 deviant and 240 standard stimuli) each were conducted, with the order of blocks counterbalanced across participants. The serial order of the stimuli was pseudo-random with one restriction: at least two standards had to occur between deviants.

The black cross at the center of screen, which was displayed throughout the stimulus blocks, unpredictably became bigger or smaller (mean frequency: 15/min; 22/block). To eliminate the potential effect peripheral stimuli might have on the outcome, participants were asked to respond as quickly and accurately as possible by pressing the left or the right button when the size of the cross changed. The response hands were counterbalanced across subjects.

### EEG Recording

EEG data were continuously recorded (bandpass 1–100 Hz, sampling rate 1000 Hz) using as NeuroLab^®^ Digital Amplifier^1^ and an electrode cap with 64 Ag/AgCl electrodes mounted according to the extended international 10-20 system and referenced to the tip of the nose. Vertical electro-oculography (VEOG) and horizontal electro-oculography (HEOG) were recorded with two pairs of electrodes; one pair was placed above and below the right eye, and the other was placed 10 mm from the lateral canthi. Electrode impedance was maintained below 5 kΩ throughout the experiment.

### ERP Analysis

Independent component analysis using Matlab R2013a (MathWorks, Inc., Natick, MA, USA) with the open-source toolbox EEGLAB (Swartz Center for Computational Neuroscience, La Jolla, CA, USA)^2^ was effectively used for EOG noise removal in the EEG. Traditionally, a digital low-pass filter at 30 Hz is applied to obtain a clean signal separated into 900-ms epochs, including a 300-ms pre-stimulus baseline time-locked to the subsequent onset of stimuli. Due to the bursts of EMG activity and amplifier clipping, affected trials were excluded from averaging. Additionally, trials contaminated by responses to changes in the fixation cross were also excluded. A total of 126 ± 15.8 and 565 ± 28.6 trials with deviant and standard stimuli, respectively, were included in the analysis. Based on analysis of the present N170 component (negative wave approximately 170 ms after the stimuli starts), P2 component (positive wave approximately 200 ms after the stimuli starts), and MMN distributions, the statistical analysis was restricted to posterior regions (P4, PO4, P6, PO6, P8, PO8, P10 and O2 over the right hemisphere and the homolog sites over the left; Amenedo et al., 2007; Kimura et al., 2009). The peak amplitudes and latencies between 120 ms and 200 ms and between 200 ms and 300 ms for N170 and P2, respectively, were measured automatically. These measures were analyzed using a repeated-measures analysis of variance (ANOVA) with stimulus type (deviant, standard), hemisphere (left, right) and site (P3/4, PO3/4, P5/6, PO5/6, P7/8, PO7/8, P9/10 and O1/2) as within-subject factors.

**Figure 2:**
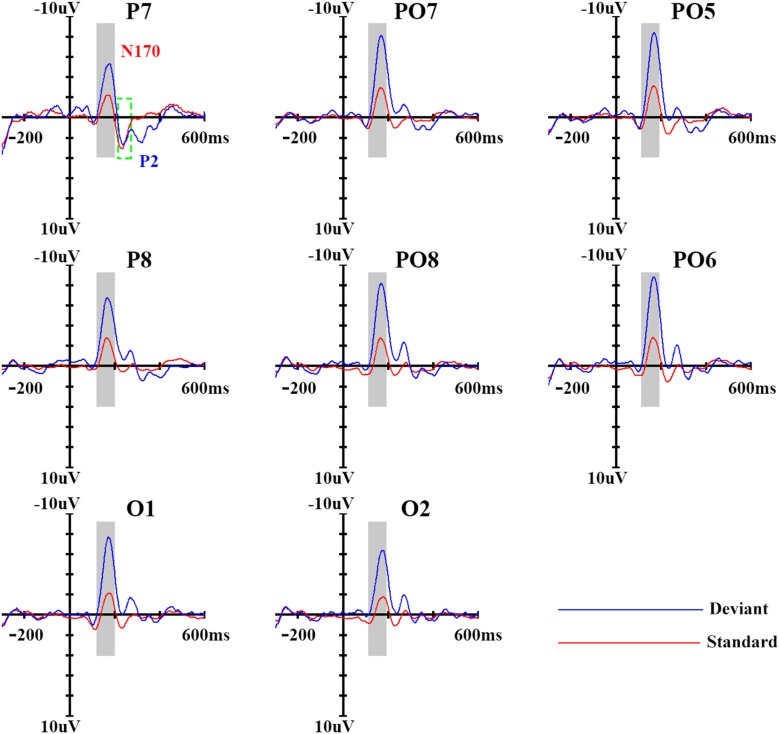
Event-related potential (ERP) waveforms of standard and deviant stimuli. Eights sites were chosen as examples; the ninth panel shows the locations of the sites, which were chosen based on previous study.

The MMN waveforms were obtained by subtracting the ERPs in response to standard stimuli from those in response to deviant stimuli. Based on the grand average MMN waveforms (see Figure 2) for each subject, the peak of the MMN components was defined as the most negative peak between 100 ms and 300 ms (based on N170 and P2 component) after stimulus onset. Subsequent visual scrutiny ensured that the most negative values represented real peaks rather than end points of an epoch. The measurements of MMN amplitudes and latencies were subjected to a repeated-measures ANOVA with hemisphere (left, right) and site (P3/4, PO3/4, P5/6, PO5/6, P7/8, PO7/8, P9/10 and O1/2) as within-subject factors. For factors with more than two levels, the degrees of freedom were corrected using the Greenhouse–Geisser procedure (for simplicity, the uncorrected degrees of freedom are presented). *Post hoc* comparisons were performed with the Bonferroni procedure.

### Time–Frequency Analysis

For assessing non-stimulus phase-locked activity, we subtracted the participant’s average response in the time-frequency domain from each individual trial, and then averaged the trials. Thus, we only created averages of the non-phase-locked spectral power (Stothart and Kazanina, 2013). Epochs were sorted according to stimulus condition to create a plot of time–frequency representations (TFRs). Total frequency band responses were analyzed via a Morlet wavelet using the Matlab wavelet toolbox (MathWorks). Morlet parameter c was set to 3 for low frequency analysis, and the final power was μV2. The TFRs of the delta band power of each participant were calculated; these ranged from 1 Hz to 4 Hz in a time window between −300 ms pre- and 600 ms post-stimulus onset, whereas the theta band ranged from 4 Hz to 7 Hz, the alpha band from 8 Hz to 13 Hz and the beta band from 15 Hz to 30 Hz. Inverse Fourier transforms were subsequently performed. We calculated the synchrony among the medial, right and left electrodes and subtracted the frequency-specific baseline (−300 to 0 ms pre-stimulus). Wavelet activity was individually returned by wavelet decomposition for each trial.

### Statistical Analysis

For all frequencies, a repeated-measures ANOVA with frequency band (delta, theta, alpha, beta), stimulus type (deviant, standard) and hemisphere (left, right) was used to examine the overall effects. For each EEG frequency band, the measurements were analyzed using a repeated-measures ANOVA treating stimulus type (deviant, standard), hemisphere (left, right) and site (P3/4, PO3/4, P5/6, PO5/6, P7/8, PO7/8, P9/10 and O1/2) as within-subject factors. For factors with more than two levels, the degrees of freedom were corrected using the Greenhouse–Geisser procedure (for simplicity, the uncorrected degrees of freedom are presented). *Post hoc* comparisons were performed with the Bonferroni procedure.

## Results

### Behavioral Data

In this study, the degree of participant attention would influence our results. So the cross that became bigger or smaller was regarded as the target cross. Subjects were asked to respond to them. Thus the response accuracy was evaluated to determine the degree of participant attention. The results showed that accuracy was more than 93.5%, which means our ERP and EEG data were recorded under unattended condition.

### ERP Data

In order to get a clear vMMN response, we used ERP analysis. As shown in Figure 2, the standard stimuli elicited N170 and subsequent P2 components at the posterior sites. Compared with standard stimuli, the deviant stimuli elicited larger N170 and smaller P2 components, representing a clear vMMN response that had a more negative deflection from 100–300 ms.

In terms of N170 amplitudes, there was a significant main effect of stimulus type (*F*_(1,14)_ = 50.30, *p* < 0.001, partial *η* = 0.81), and deviant stimuli elicited a larger N170 component (−7.7 μV) than did standard stimuli (−3.2 μV). The main effect of site was also significant (*F*_(3,42)_ = 5.83, *p* < 0.05, partial *η* = 0.29), revealing a partial lateral distribution of N170 amplitudes. The ANOVA of N170 peak latencies showed that deviant stimuli were associated with longer N170 latencies (169 ms) compared with standard stimuli (164 ms; *F*_(1,14)_ = 15.60, *p* < 0.01, partial *η* = 0.56). No other main effects or interactions were significant (*p* > 0.1).

A similar ANOVA analysis was conducted for the P2 component. We found a significant main effect of stimulus type (*F*_(1,14)_ = 17.40, *p* < 0.01, partial *η* = 0.59), indicating that deviant stimuli elicited a smaller P2 component (2.0 μV) than did standard stimuli (3.80 μV). With regard to P2 peak latencies, the ANOVA revealed a significant main effect of stimulus type (*F*_(1,14)_ = 10.77, *p* < 0.05, partial *η* = 0.47), indicating that deviant stimuli were associated with longer P2 latency (258 ms) compared with standard stimuli (236 ms). No other main effects or interactions were significant for the P2 component (*p* > 0.1).

**Figure 3:**
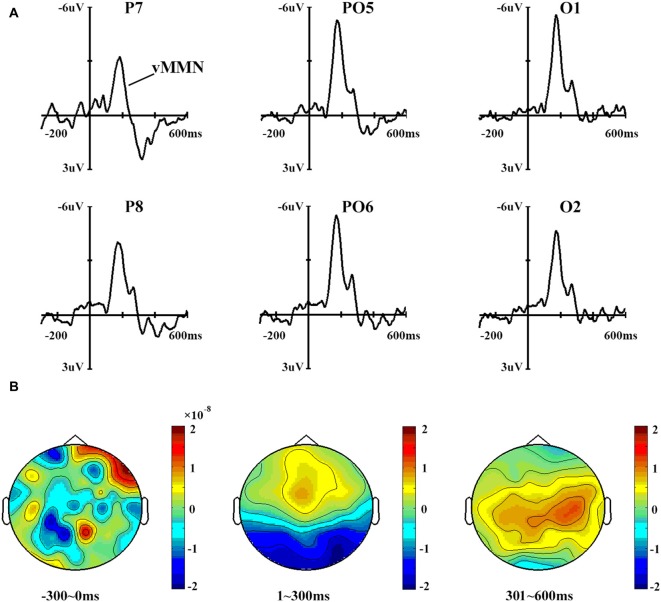
**(A)** Mismatch negativity (MMN) waveforms obtained by subtracting the ERPs in response to standard stimuli from those in response to deviant stimuli. Six sites were chosen as examples. **(B)** The topographical distribution of MMN, with time windows of 0–300 ms, 301–600 ms and 601–900 ms.

Figure 3 shows the vMMN waveforms and topographical distribution of vMMN. A one-way ANOVA showed that site had a significant effect on vMMN peak amplitudes (*F*_(3,42)_ = 2.996, *p* < 0.05) but not on latencies (*p* > 0.1).

### Time–Frequency Analysis

**Figure 4:**
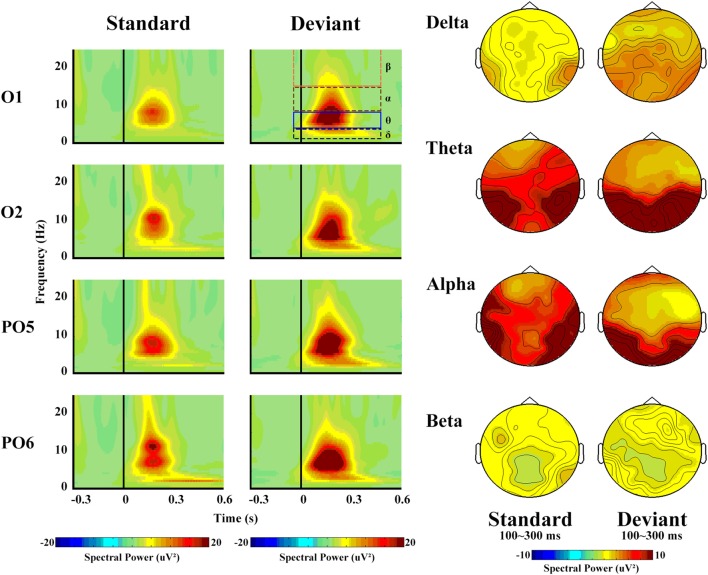
The left panel shows the spectral powers of different frequencies of standard and deviant stimuli. Four sites were chosen as examples. The right panel shows the topographical distribution of different frequencies for standard and deviant stimuli, with a time window of 100–300 ms.

From the view of frequency domain, we could better know about vMMN. Figure 4 shows the spectral activity of deviant and standard stimuli. The oscillation power occurred primarily between 100 ms and 300 ms, which is similar to the time window of the vMMN.

The spectral power revealed a clear difference in the frequency band, with maximum theta (14.49 μV2) and minimum beta (1.99 μV2, *F*_(3,42)_ = 26.55, *p* < 0.001, partial *η* = 0.92). Deviant stimuli were more powerful (8.57 μV2) than standard ones (6.13 μV2, *F*_(1,14)_ = 7.06, *p* = 0.03, partial *η* = 0.44), but no significant differences with regard to hemisphere or site were observed.

In terms of the delta band, only the main effect of stimulus type was significant (*F*_(1,14)_ = 76.91, *p* < 0.001, partial *η* = 0.90), as a greater increase in delta spectral power was observed in response to deviant (5.40 μV2) than to standard (3.14 μV2) stimuli.

**Figure 5:**
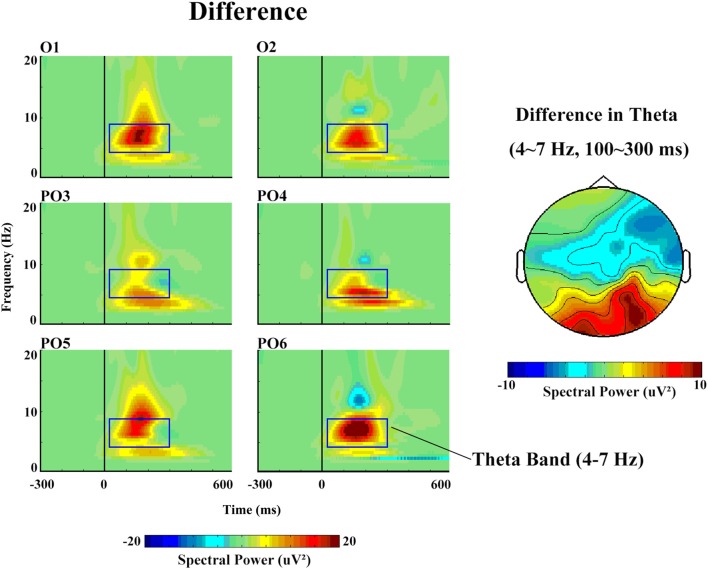
The spectral power and topographical distribution of differences in theta band activity. The difference refers to the spectral power in response to deviant stimuli minus that in response to standard stimuli.

With respect to the theta band (Figure 5), the ANOVA revealed a main effect of stimulus type (*F*_(1,14)_ = 5.85, *p* = 0.04, partial *η* = 0.40), as a greater increase in theta spectral power was observed in response to deviant (17.57 μV2) than to standard (11.41 μV2) stimuli.

The same type of ANOVA analysis revealed no significant differences in the alpha and beta bands. However, the alpha band reflected a greater increase in power in response to deviant (9.46 μV2) than to standard (7.84 μV2) stimuli, whereas the beta band reflected the opposite pattern (2.14 μV2 in response to standard and 1.85 μV2 in response to deviant stimuli).

## Discussion

The aim of this study is to explore the vMMN related to the automatic detection of directional changes. We focused on the cardinal orientations, and the arrow symbol was used as the stimuli in this study. The EEG data were divided based on four oscillatory frequencies: delta, theta, alpha and beta. For each frequency band, the non-phase-locked spectral power was calculated independently. The results confirmed that changes in orientation under unattended condition could induce vMMN. In addition, our results further indicated that deviant stimuli could induce stronger spectral power in delta and theta bands.

In line with previous studies, deviant stimuli elicited larger N170 and smaller P2 components compared with standard stimuli. Based on the differences, we found that the participants could notice the orientation changes (0° and 90°/270°) easily. However, whether they could notice the arrow change (90°/270°) is not clear. Recently, researchers used arrow stimuli to find early attention direction negativity effects 220–260 ms after the arrow stimulus onset (Hietanen et al., 2008). Compared with our ERP results, we can infer that participants did not notice the arrow (we did not find an early attention direction negativity effect). Participants only noticed the orientation changes.

MMN is usually obtained through ERP analysis when an unexpected event occurs. It is analyzed by subtracting the ERP waveforms in response to standard stimuli from those in response to deviant stimuli. vMMN could be induced by changes in many kinds of object characteristics. Our study agrees with previous studies that showed that changes in orientation can induce vMMN (Kimura et al., 2009; Stefanics et al., 2014).

The time–frequency analysis showed that deviant stimuli elicited enhanced band power compared with standard stimuli; this was the case for the delta and theta bands, which may be attributable to the lower probability and novelty of deviant stimuli and which may have been influenced by principles of perception (Parmentier et al., 2011a,b). Replicating one recent report on auditory MMN (aMMN) response (Stothart and Kazanina, 2013), we also found that deviant stimuli induced an increase in theta power before 25–300 ms post-stimulus onset. These data indicate that theta activity appears to play a role in the generation of the vMMN response (Stothart and Kazanina, 2013). Thus, both aMMN and vMMN are related to theta bands. We can infer that MMN is more associated with theta response. The present study found the greatest theta activity (i.e., enhanced theta oscillation) in response to deviant vs. standard stimuli, indicating that theta activity may play an important role in cognitive processes involving automatic change detection.

Although we also observed increased delta power in response to deviant vs. standard stimuli, we did not consider it correlated with MMN because delta oscillations constitute one of the major operating rhythms of the P300 component (Başar et al., 2001), which has historically been viewed as an important ERP component elicited by infrequent target stimuli in the auditory oddball paradigm (Öniz and Başar, 2009). However, this component was less evident in vMMN, and the P300 may be the main difference between aMMN and vMMN, but this possibility requires further investigation. Additionally, delta oscillations contribute to making a decision and detecting a signal (Başar et al., 2001). Therefore, delta response did not correlate with MMN. In some studies of MMN, researchers found changed post-auditory stimulus alpha/beta power using an oddball paradigm (Hsiao et al., 2009; Öniz and Başar, 2009; Stothart and Kazanina, 2013; Başar et al., 2015). However, in this study, we did not find a correlation between vMMN and alpha/beta bands. This difference may be caused by the different tasks.

In summary, to investigate the EEG oscillatory characteristics of vMMN, we analyzed ERP and EEG data elicited by standard (0° arrows) and deviant (90°/270° arrows) stimuli. Compared with standard stimuli, deviant stimuli elicited larger negative N170 and smaller P2. In addition, changes in orientation could induce vMMN as expected. According to the time–frequency analysis, deviant stimuli elicited enhanced band power compared with standard stimuli in delta and theta bands. Furthermore, theta activity played an important role in the generation of the vMMN induced by changes in orientation. Examination of both ERPs and EEG oscillation provides a more complete picture of the event-related changes in vMMN responses.

However, the present study has some limitations. For instance, the sample was small, although the present method of statistical analysis is reliable. It is necessary to further investigate the present results in a clinical evaluation of cognitive function using a larger sample size.

## Author Contributions

TY and LZ designed experiments; ZL, XT and TQ carried out experiments; NM and YF analyzed experimental results. TY, TL, LW and XD wrote the manuscript.

## Conflict of Interest Statement

The authors declare that the research was conducted in the absence of any commercial or financial relationships that could be construed as a potential conflict of interest.

## Footnotes

^1^www.brainstarbj.com

^2^http://sccn.ucsd.edu/eeglab/
